# Upregulation of Glutamate Transporter 1 by Clavulanic Acid Administration and Attenuation of Allodynia and Hyperalgesia in Neuropathic Rats

**DOI:** 10.32598/bcn.10.4.799.2

**Published:** 2019-07-01

**Authors:** Bahareh Amin, Mahmoud Avaznia, Reihaneh Noorani, Soghra Mehri, Hossein Hosseinzadeh

**Affiliations:** 1. Cellular and Molecular Research Center, Department of Physiology and Pharmacology, School of Medicine, Sabzevar University of Medical Sciences, Sabzevar, Iran.; 2. Pharmacodynamics and Toxicology, School of Pharmacy, Mashhad University of Medical Sciences, Mashhad, Iran.; 3. Pharmaceutical Research Center, Pharmaceutical Technology Institute, Mashhad University of Medical Sciences, Mashhad, Iran.

**Keywords:** Clavulanic Acid, Chronic constriction injury, Glutamate Transporter 1

## Abstract

**Introduction:**

Clavulanic acid (CLAV) is structurally similar to ceftriaxone, a potent stimulator of glial GlutamateTransporter-1 (GLT-1) expression. The present study aims at exploring the anti-nociceptive effects of CLAV, a beta-lactamase inhibitor in rats underwent sciatic nerve Chronic Constriction Injury (CCI).

**Methods:**

CLAV (12.5, 25, 50 mg/kg) was administered intraperitoneally after the surgery for 14 consecutive days. Behavioral pain parameters were evaluated before and 3, 5, 7, 10 and 14 days after injury. Spinal GLT-1 level was measured via western blotting at days 7 and 14.

**Results:**

CCI led to mechanical allodynia, cold allodynia and thermal hyperalgesia which started on postoperative days 3 and continued until the end of study. We found that CLAV (12.5 and 25 mg/kg) significantly attenuated all pain related behaviors as compared to the CCI animals treated with normal saline. Protein level of GLT-1 was down-regulated on day 14 following CCI and this phenomenon was reversed by fourteen days treatment of CLAV at the low doses of 12.5 and 25 mg/kg.

**Conclusion:**

These results suggest that CLAV might provide a new therapeutic strategy for neuropathic pain and its effect might be partially associated with the up-regulation of GLT-1.

## Highlights

- Clavulanic acid showed anti-allodynic and anti-hyperalgesic effects in animal models with neuropathic pain.
- GLT1 protein decreased in the spinal cord of neuropathic animals.
- The antinociceptive effects of clavulanic acid in neuropathic rats depend on GLT-1 up-regulation.

## Plain Language Summary

Neuropathic pain is a challenge in clinical practice. The recommended drugs for the pain have many side effects with limited efficacy. Clavulanic Acid (CLAV) is a member of β-lactam antibiotics, such as penicillins and cephalosporins. It has been reported that CLAV has neuroprotective effects in some studies. In this work, chronic constriction injury (CCI) of sciatic nerve induced increased sensitivity to mechanical and cold stimuli. Administration of CLAV immediately after the injury could attenuate pain developed in CCI animals. Based on our results, glutamate transporter (GLT1) content decreased in CCI animals treated with normal saline. This protein is responsible for preventing the toxicity of glutamate to brain cells. The level of GLT1 was high after intraperitoneal administration of CLAV in the lumbar spinal cord of CCI rats.

## 1. Introduction

Nerve-injury-induced neuropathic pain is characterized by spontaneous pain, allodynia (when normally innocuous stimuli become painful) and hyperalgesia (when sensitivity to painful stimuli increases). A variety of undesirable side effects of prototypical drugs makes this problem a significant challenge in clinical practice (Dworkin et al., 2010). Therefore, discovering and developing new drugs provide a new way to treat refractory neuropathic pain.

Spinal cord glutamate has been reported to play a critical role in the development of hyperalgesia following nerve injury, by activating various glutamate receptors (Coderre, Kumar, Lefebvre, Yu, 2007; Zhang, Chen, Pan, 2009). Additionally, there is a link between glutamate transporters downregulation and chronic pain conditions (Tao, Gu, Stephens, 2005). β-Lactam antibiotics enhance cellular glutamate uptake via spinal upregulation of Glutamate Transporter subtype 1 (GLT-1). GLT1 is the predominant astrocytic transporter, responsible for about 90% of glutamate uptake in the brain (Hu et al., 2010; Ramos et al., 2010).

The representative β-lactam antibiotic, ceftriaxone, has shown neuroprotective effects in some neurodegenerative diseases such as amyotrophic lateral sclerosis, multiple sclerosis, stroke, depression, tolerance, addiction, as well as neuropathic pain (Amin, Hajhashemi, Hosseinzadeh, & Abnous, 2012; Chen, He, & Wang, 2012; Rothstein et al., 2005).

However, ceftriaxone has little therapeutic value because of concerns about resistance development to antibiotic (Kaplan & Mason, 1998). Another limitation of ceftriaxone is poor brain penetrability due to water-solubility of this compound that requires the administration of high doses of ceftriaxone, leading to increased risk of adverse effects. Ceftriaxone also needs parenteral administration, which is a painful and costly route, diminishing patient’s compliance (Friedland, Kulick, Biro, & Patterson, 1996).

Clavulanic Acid (CLAV) is a member of β-lactam antibiotics, such as penicillins and cephalosporins. However, this drug has weak antibacterial activities and consequently, no therapeutic efficacy. CLAV acts as an irreversible inhibitor of bacterial β-lactamase enzymes that naturally degrade and inactivate β-lactam antibiotics. Due to this effect, CLAV has been commonly used in combination with some β-lactam antibiotics such as ticarcillin and amoxicillin to overcome β-lactamase-mediated resistance (Crosby & Gump, 1982). CLAV readily penetrates the blood-brain barrier (Nakagawa, Yamada, Tokiyoshi, Miyawaki, & Kanayama, 1994). This drug is orally stable and effective, with the bioavailability of approximately 64% to 75% (Bolton, Allen, Davies, Filer, & Jeffery, 1986).

It has been reported that CLAV has neuroprotective effects to prevent or reduce neuronal damage in patients suffering from or susceptible to disease conditions characterized by loss of neuronal cells or loss of neuronal cell function (Slusher, Jackson, Paul, Tays, Maclin, 2000). CLAV has been shown anti-convulsion (Chen et al., 2013), antidepressant, anxiolytic effects (Kim et al., 2009), plus stimulatory effect on sexual behaviors (Chan, Kim, Ahn, Oosting, & Olivier, 2009). Also, it protects against neurodegenerative Parkinson and Alzheimer diseases (Huh et al., 2010) as well as attenuation of morphine’s tolerance, rewarding, hyperthermia, and locomotor-sensitizing actions (Schroeder et al., 2014).

Anti-nociceptive and anti-inflammatory effects of acute administration of CLAV have been demonstrated in acetic acid-induced writhing, formalin-induced pain, as well as carrageenan-induced paw edema (Banani et al., 2012; Hajhashemi & Dehdashti, 2014). However, the effect of this drug and possible mechanisms of its action have not yet been evaluated in the chronic conditions of pain, such as peripheral neuropathic pain. The present study was undertaken to evaluate the antinociceptive effects of repeated administration of CLAV, intraperitoneally, in the Chronic Constriction Injury (CCI) model of neuropathic pain in rats.

Considering that neuropathic pain is associated with the decreased expression of glutamate transporters, drugs that increase the levels of this protein are effective in the treatment of neuropathic pain syndrome (Mao & Yang, 2010; Ramos et al., 2010). In this study, we wanted to find out whether β-lactamase inhibitor, CLAV, displays antinociceptive effects through Glutamate Transporter 1 (GLT1) activation or not. The protein levels of GLT-1 were evaluated via western blotting on days 7 and 14 after an operation in CCI animals treated with normal saline or CLAV.

## 2. Methods

### 2.1. Study materials

CLAV was donated by Daana Pharmaceutical Co. (Tabriz, Iran). It was dissolved in Normal Saline (NS). The solution was administered Intraperitoneally (IP) at the doses of 12.5, 25, and 50 mg/kg. Doses of CLAV in the present study were comparable to the doses reported in the literature (Banani et al., 2012). Sodium pentobarbital was purchased from Claris Lifesciences CO. (India), dissolved in NS and injected at a dose of 100 mg/kg IP.

### 2.2. Study animals

Male Wistar rats, weighing 250 to 270 g at the start of surgery, were obtained from the animal center of School of Pharmacy, Mashhad University of Medical Sciences, Iran. The animals were maintained on a 12:12 light:dark cycle at 22°C with food and water ad libitum. Behavioral studies were carried out in a quiet room between 9:00 AM and 11:00 AM. The animals in the present study were cared for and used following the principles and guidelines outlined by Internationally Accepted Principles for Laboratory Animal Use and Care, to minimize pain or discomfort in animals (Zimmermann, 1983). All experimental protocols were approved by the Mashhad University of Medical Sciences, Mashhad, Iran (approval number: 910634).

### 2.3. Chronic constriction injury

Mononeuropathy was induced as described previously by the method of Bennet and Xie (Bennett, Chung, Honore, Seltzer, 2003). The rats were anesthetized with sodium pentobarbital (60 mg/kg, IP). The sciatic nerve was exposed at mid-thigh and freed of connective tissue. Then, four chromic gut sutures (4-0) were loosely tied around the nerve with a 1.0–1.5 mm interval between each of them. The wound was closed in layers with silk sutures (4-0).

### 2.4. Experimental protocol

#### 2.4.1. Animals were divided into the following groups

Group I: Naive animals (n=6); Group II: Sham group: In this group, rats exposed to similar surgical conditions except for nerve ligation and treated with the high dose of Clavulanic Acid (50 mg/kg) (n=9); Group III: Rats subjected to a Chronic Constrictive Injury (CCI), were injected with normal saline once daily for 14 days, which began immediately after injury and were designated to be the control group (n=9); Groups IV, V and VI: CCI+IP administration of CLAV (12.5, 25, and 50 mg/kg) in rats, respectively, once daily for 14 days, which began immediately after injury (n=9).

Behavioral testing was performed in all animals one day before the operation (day-1), and subsequently on days 3, 5, 7, 10, and 14 after the sciatic nerve CCI. To determine the time course of spinal GLT1s changes after CCI, on the postoperative day 7, or 14 after the end of behavioral tests, three separate animals in each group were harvested, and their lumbar spinal cord sections were dissected. The samples were kept in the individual tubes, quickly frozen in liquid nitrogen and then stored at −80°C until they were used.

### 2.5 Nociceptive behavior tests

#### 2.5.1. Measurement of mechanical allodynia

Mechanical allodynia was measured by the calibrated von Frey filaments (Stoelting, Wood Dale, IL, USA). Rats were placed in individual transparent Perspex cubicles with a wire mesh bottom, to get familiar with the environment. The filaments of sequentially increasing stiffness were applied to the plantar surface of the hind paw of animals. The force needed to elicit animal withdrawal was expressed as the mechanical Paw Withdrawal Threshold (PWT) in grams (g) (Bennett et al., 2003).

#### 2.5.2. Measurement of cold allodynia

Fifteen minutes after the end of mechanical allodynia test, acetone drop was used to the plantar surface of the injured hind paw to assess cold allodynia. Acetone was applied five times to the hind paw, with a gap of 5 min between the applications and a sudden withdrawal response to the acetone was considered as a sign of cold allodynia. The frequency of paw withdrawal was expressed as a percentage (the number of paw withdrawals divided by the total number of trials times 100) (Yoon, Wook, Sik, Ho, & Mo, 1994).

#### 2.5.3. Measurement of thermal hyperalgesia

Thermal hyperalgesia was assessed using a Plantar Test Apparatus (Ugo Basile, Varese, Italy). The animals were acclimatized in the plastic cage of apparatus for about 30 min. The plantar surface of the hind paw was exposed to a beam of radiant heat through the glass floor. Three latency measurements were taken and averaged for each hind paw for each session of testing. The cut-off time was 30 s to prevent tissue damage (Hargreaves, Dubner, Brown, Flores, & Joris, 1988).

### 2.6. Western blot

At respective time points (day 7 and 14 post-CCI) spinal cord samples of sham, NS-CCI animals, and CCI animals treated with CLAV at different doses of 25, 50 and 100 were homogenized in the lysis buffer (50 mM Tris-HCl, pH: 7.4, 2 mM EDTA, 2 mM EGTA, 10 mM NaF, 1 mM sodium orthovanadate (Na_3_VO_4_), 10 mM β-glycerophosphate, 0.2% W/V sodium deoxycholate, 1 mM phenylmethylsulfonyl fluoride, and complete protease inhibitor cocktail) (Roche, Mannheim, Germany).

The homogenate was then sonicated on ice with three bursts at high intensity lasted 10-s, with a 10-s cooling period between each burst. In the end, homogenized tissues were centrifuged at 10000g for 10 min at 4°C, and proteins were quantified using the Bradford assay kit (BioRad) and adjusted (Bradford, 1976). The samples were electrophoresed through SDS-polyacrylamide gels and transferred to PVDF membranes. Non-fat milk in TBS (5%) was used to block blots for one hour before incubation with antibodies.

Primary antibodies utilized in this study, were as follows: rabbit monoclonal anti-serum against GLT-1 (Cell Signaling#3838) and rabbit polyclonal anti-serum against β-actin (Cell Signaling#4967). After washing three times with TBST, the blots were probed with HRP-conjugated antibodies (Cell Signaling#7074) and developed with Enhanced Chemiluminescence (ECL, USA) reagents. Alliance 4.7 Gel doc (UK) was used to visualize the peroxidase-coated bands. Bands of proteins were densitometrically quantified using UVtec software (UK).

### 2.7. Data analysis and statistics

The results were presented as the Mean±SEM. Repeated-measures Analysis of Variance (ANOVA) tests for behavioral studies were done with a group (between-subjects factor) and time after nerve ligation (within-subjects factor). The Bonferroni test was examined post hoc for multiple comparisons at individual time points between groups. The data obtained from the western blot-test were analyzed by 1-way ANOVA, followed by Tukey’s test. For all tests, P<0.05 was considered statistically significant.

## 3. Results

Per se administration of CLAV (50 mg/kg IP) to sham animals did not change behavioral parameters as compared to naive ones (data not shown).

### 3.1. The influence of Clavulanic Acid on the CCI-induced development of mechanical allodynia

**Figure 1.**
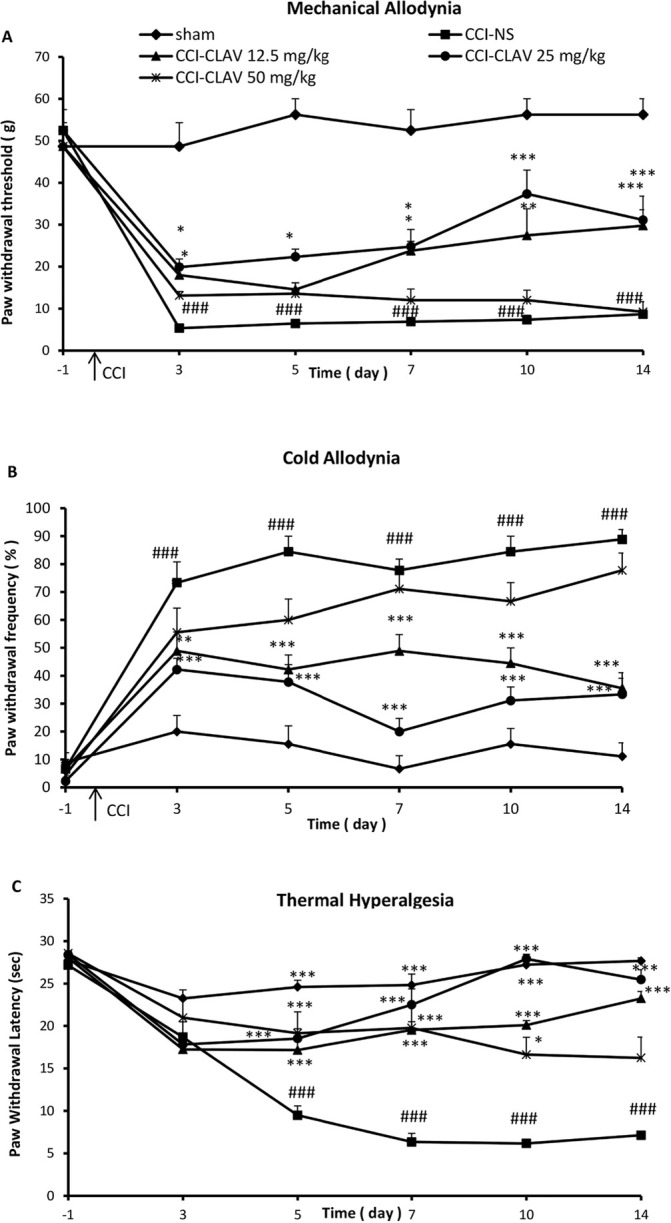
Effects of Clavulanic Acid (CLAV) on the neuropathic pain induction A: Mechanical allodynia; B: Cold allodynia; and C: Thermal hyperalgesia after Chronic Constriction Injury (CCI) to the sciatic nerve. Treatment was performed with an intraperitoneal injection of 12.5, 25, and 50 mg/kg of CLAV, once daily for 14 days beginning immediately after the operation. Values are presented as mean±SEM, n=9 rats per group; The presented data were analyzed by 2-way ANOVA with repeated measures, followed by Bonferroni Post Hoc Test. ^*^P<0.05, ^**^P<0.01 and ^***^P<0.001 as compared to CCI rats treated with normal saline (NS). ^###^P<0.001 indicates a comparison between CCI-NS and sham animals.

Before operation (day-1), there was no significant difference in the paw withdrawal threshold to tactile stimuli (mechanical allodynia) in CCI group (52±5.3 g) as compared with sham animals (48.6±5.6 g). Decreased response threshold to the mechanical allodynia developed 3 days after loose ligation of the sciatic nerve as compared to sham animals (5.3±0.75 g and 48.5±5.7 respectively; P<0.001) (Figure. 1A). The high dose of 50 mg/kg of CLAV failed to attenuate hypersensitivity to von Frey filaments at all days of study. Administration of CLAV (12.5 and 25 mg/kg) attenuated CCI-induced decrease in the paw withdrawal threshold on postoperative days 3, 5, 7, 10 and 14 as compared to normal saline treated group. Figure 1A shows the time course of the increase in the paw withdrawal threshold produced by CLAV in CCI animals.

### 3.2. The influence of Clavulanic Acid on the CCI-induced development of cold allodynia

There was no significant difference in the paw withdrawal frequency to acetone drop (cold allodynia) in CCI animals (6.7±7.3%) as compared with the sham-operated group (8.8±3.5%), before the operation (day-1). Rats receiving constriction of the left sciatic nerve developed robust cold allodynia as early as day 3 post-CCI (73±7.4%) that lasted throughout the study. In contrast, the threshold for the sham animals remained unchanged (20±5.7%) (P<0.001, Fig. 1B). Intraperitoneal treatment with CLAV (12.5, 25 mg/kg) except the high dose of 50 mg/kg for 14 days reversed nerve injury-induced cold allodynia on days 3, 5, 7, 10 and 14. Figure 1B shows the time course of decrease in the paw withdrawal frequency due to the administration of CLAV in CCI animals.

### 3.3. The influence of Clavulanic Acid on the CCI-induced development of thermal hyperalgesia

Before the operation, the mean paw withdrawal latency in CCI rats (27.2±1.3 s) was not significantly different from sham animals (28±1.5 sec.). Five days after the operation, the neuropathy induced by CCI resulted in a significant development of thermal hyperalgesia (9.4±1.8 s) as compared to the sham control group (24±1.4 s) (P<0.001) which continued on postoperative days 7, 10 and 14 in normal saline-treated CCI rats (Figure. 1C). The rats were concomitantly administered with CLAV (12.5 and 25 mg/kg) for 14 days significantly attenuated such decreased mean paw withdrawal latency as compared with NS-CCI animals. CLAV at the dose of 50 mg/kg attenuated hypersensitivity to thermal stimulus but was not able to retain such effect until the end of the study. Figure 1C shows the time course of the increase in the paw withdrawal latency produced by CLAV.

### 3.4. The influence of Clavulanic Acid on the CCI-induced changes in GLT1

**Figure 2.**
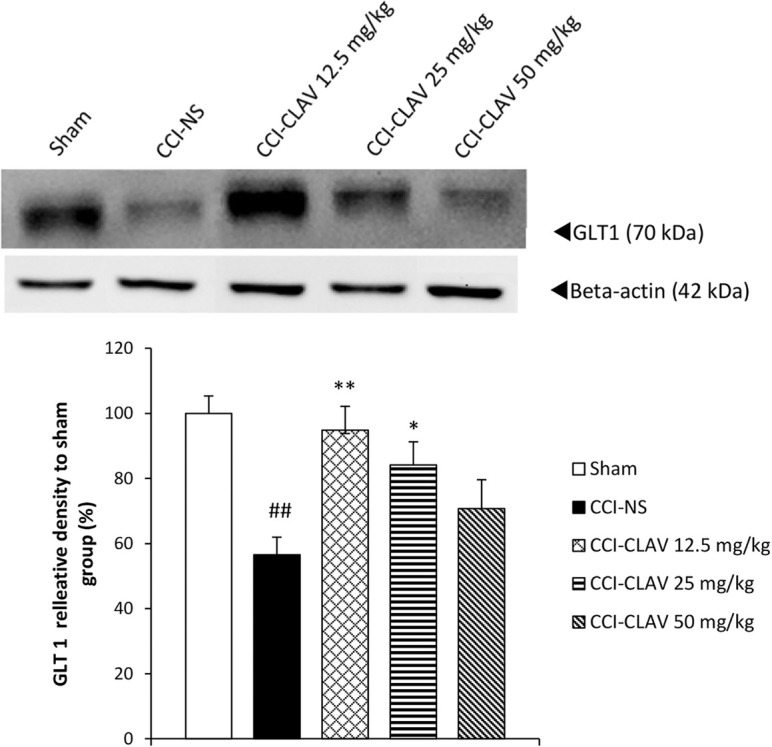
Effect of Clavulanic Acid (CLAV) on the spinal glutamate transporter 1 (GLT-1) protein level of CCI rats A. Effect of Clavulanic Acid (CLAV) on the spinal glutamate transporter 1 (GLT-1) protein level of CCI rats in western blotting analysis. CLAV was administered at doses of 12.5, 25, and 50 mg/kg IP, once daily for 14 days beginning immediately after the operation. Beta-actin is the loading control protein. B. Represents a quantitative presentation of the immunoblots. Values have been shown as the Mean±SEM (n=3). The data were analyzed by 1-way ANOVA followed by Tukey test for pair-wise comparison. ^*^P<0.05; ^**^P<0.01 as compared with CCI rats treated with normal saline; and ^##^P<0.01 indicates a comparison between CCI-NS and sham animals.

The results of western blot analysis are shown in Figure 2A and 2B. When compared with the sham rats, the protein levels of GLT1 decreased on day 7, but not to a significant extent (data not shown). A significant increase was detected in the lumbar spinal cord levels of GLT1 protein in CCI rats, on day 14(P<0.01). CLAV at the low doses of 12.5 mg/kg (P<0.01) and 25 mg/kg (P<0.05) increased the GLT1 levels of spinal cord in CCI animals.

## 4. Discussion

CCI of the sciatic nerve is induced mechanical allodynia and cold allodynia. It started from postoperative day 3 and lasted up to day 14. Fourteen days administration of CLAV (12.5 and 25, but not 50 mg/kg IP), beginning immediately after the injury could attenuate mechanical allodynia and cold allodynia developed in CCI animals compared to NS-CCI animals. Thermal hyperalgesia started with latency on day 5 after CCI. CLAV at three applied doses decreased hyperalgesia to thermal radiant heat stimulus; however, the effect of 50 mg/kg of CLAV did not remain until day 14.

In Kim et al. (2009) study, seven days administration of low dose CLAV showed greater anxiolytic effect. An inverted-U shaped dose-response might be responsible for such profile of CLAV’s dose-response, which is called hormetic response (Calabrese & Baldwin, 2001). We hypothesized here that CLAV a structurally related β-lactamase inhibitor would also influence the GLT1 expression. To this end, we assessed the effects of repeated CLAV administration on the protein levels of GLT1 on days 7 and 14 post-CCI.

Based on our results, GLT1 protein content decreased in CCI animals treated with normal saline on postoperative day 7 but not to a significant degree, as compared to the sham group. However, on day 14, levels of protein significantly decreased in the NS-CCI spinal cord of animals. In a study conducted on Sprague Dawley CCI rats, Sung et al. found a biphasic change in the GLT-1 level after CCI; GLT1 was upregulated on days 3 and 4, followed by a decrease on postoperative days 7 and 14 (Sung, Lim, & Mao, 2003).

The observed discrepancies might be attributed to species difference and methods used. The level of GLT1 rose on day 14 after intraperitoneal administration of CLAV at low doses of 12.5 and 25 but not the high dose (50 mg/kg) in the lumbar spinal cord of CCI rats. Considering that hyperalgesia was attenuated by CLAV 50 mg/kg, it might be postulated that other pathways had a more important role in the induction of thermal hyperalgesia. Although the time course of GLT-1 upregulation by Clavulanic Acid was not investigated on days 1 and 3 after CCI, it would be the focus of future research.

Our data on the upregulation of GLT-1 are supported by Rawls et al., (2010) results reporting that GLT-1 transporter inhibitor Dihydrokainite (DHK) augmented seizure-like activity induced by glutamate and cocaine. In the study of Schroeder et al. (2014), CLAV administration (10 mg/kg) showed a reduction in rewarding and sensitizing effects of morphine, similar to ceftriaxone at the dose 200 mg/kg.

Neuroprotective effects of β-lactam antibiotics are principally dependent on the GLT1 upregulation and are perhaps concentrated on the β-lactam ring itself (Konaklieva, Plotkin, & Herbert, 2009). Ceftriaxone, Clavulanic Acid, and tazobactam prevented the seizure-like activity induced by glutamate or cocaine administration in planarian, whereas vancomycin, an antibiotic, which lacks the β-lactam ring, was not active in this assay. In the screening study of Rothstein et al. (2005), non-β-lactam antibiotics, including kanamycin, fluconazole, minocycline, polymyxin, and doxycycline, did not affect GLT1 protein expression.

Another reason supporting GLT-1 possible role in the antinociceptive effects of Clavulanic Acid is that the acute administration of this drug fails to show anti-seizure activity in three models of seizure of 6-Hz seizure threshold, Maximal Electroshock Seizure Threshold (MEST) test, and Intravenous Pentylenetetrazole (IV PTZ injection) seizure. Sexual stimulating activity observed with the acute administration of a high dose of Clavulanic Acid is less than that obtained with the low dose in chronic administration (Rawls et al., 2010). Therefore, the 7-day administration of ceftriaxone is required for ceftriaxone to increase the protein level of GLT-1 in rats’ brains (Rothstein et al., 2005). However, the contribution of other pathways in the antinociceptive effects of CLAV is also possible.

The supra-spinal dopaminergic system is also related to the suppression of tonic pain. In a study by Coffeen et al. (2010), inflammation induced by carrageenan decreased extracellular levels of dopamine in the cortex of rats, correlated with the reduced paw withdrawal latency in the plantar test. Furthermore, L-Dopa decreased pain in diabetic polyneuropathy patients (Ertas, Sagduyu, Arac, Uludag, & Ertekin, 1998). Clavulanic Acid treatment protected hippocampal and dopamine neurons in kainic acid and 1-Methyl-4-Phenyl-1,2,3,6-Tetrahydropyridine (MPTP) rodent models displaying the characteristics of idiopathic Alzheimer and Parkinson disease, respectively, with improving the MPTP-induced motor deficits.

Sanna et al. (2011) reported that clavulanic acid induced penile erection and yawning in male rats by increasing the serotonin and dopamine neurotransmission. In an in vitro study conducted on the PC12 and SH-SY5Y cells, Clavulanic Acid enhanced release of dopamine in PC12 and SH-SY5Y cells without affecting dopamine synthesis (Kost et al., 2011).

Also, the development of neuropathic pain may be associated with the activation of apoptotic events (Siniscalco et al., 2007). Bax, as an apoptotic factor and Bcl-2 an anti-apoptotic protein in the Bcl-2 family, are responsible for the subsequent activation of caspases and mitochondrial-mediated apoptosis (Keane et al., 2001).

Kost et al. (2012) investigated the anti-apoptotic property of Clavulanic Acid. A significant increase in the mitochondrial membrane potential of cells treated with Clavulanic Acid was observed in cells incubated with neurotoxin 1-Methyl-4-Phenylpyridinium (MPP+), as a model of Parkinson disease. Increased levels of Bax and cytochrome C, as well as caspases 3 and 9 activations induced by MPP+, decreased in Clavulanic Acid-treated cells. Anti-apoptotic Bcl-xl protein became also normalized. However, it is not the only mechanism, and CLAV may act through multiple mechanisms.

In summary, our data show that CLAV displays antiallodynic and anti-hyperalgesic effects in sciatic nerve CCI rats. Increased protein levels of GLT-1 contribute, at least in part, to the antinociceptive effects obtained with this drug. The property that Clavulanic Acid possesses little intrinsic anti-bacterial activities strengthens its application for further therapeutic development. Structural similarities between CLAV and ceftriaxone and the more favorable pharmacokinetic of CLAV suggest that this drug has the ability for further studying the management of chronic pain conditions.
